# The role of pharmacogenomics in the discontinuation of psychotropic medication: a scoping review protocol

**DOI:** 10.12688/hrbopenres.14314.1

**Published:** 2026-01-05

**Authors:** Natalia Stollarova, Cristín Ryan, Greg Sheaf, Dolores Keating, Brian O’Donoghue, Cathal Cadogan

**Affiliations:** 1 School of Pharmacy and Pharmaceutical Sciences, Trinity College Dublin, Dublin, Ireland; 2 Department of Pharmacology and Toxicology, Faculty of Pharmacy, Comenius University Bratislava, Bratislava, Slovakia; 3 The Library of Trinity College, Trinity College Dublin, Dublin, Ireland; 4 Pharmacy Department, Saint John of God University Hospital, Dublin, Stillorgan, Ireland; 5 Department of Psychiatry, School of Medicine, University College Dublin, Dublin, Ireland

**Keywords:** Pharmacogenomics, Psychotropic medications, Discontinuation, Withdrawal, Tapering, Precision medicine, Scoping review

## Abstract

**Introduction:**

Discontinuation of psychotropic medication is associated with considerable clinical challenges, including withdrawal symptoms and relapse. Pharmacogenomic testing is increasingly used in clinical practice to optimise pharmacotherapy by guiding drug selection and dosing, however, its role in informing medication discontinuation and tapering strategies remains underexplored. Understanding the influence of genetic variability on withdrawal symptoms could support safer, individualised discontinuation practices.

**Objective:**

This scoping review aims to map the available evidence on the role of pharmacogenomics on the discontinuation of psychotropic medications.

**Methods:**

The review will be conducted in accordance with the Joanna Briggs Institute (JBI) methodology for scoping reviews and reported using the PRISMA-ScR checklist. Comprehensive searches will be performed in MEDLINE, EMBASE, CINAHL, Web of Science, and PsycINFO. Studies exploring gene polymorphisms relevant to the discontinuation of psychotropic medications (antidepressants, antipsychotics, mood stabilisers, anxiolytics, hypnotics, stimulants, and opioids) will be considered. Following screening, data will be extracted, and a narrative synthesis will be undertaken to map characteristics of included studies, genes studied, medication classes involved, reported outcomes, and knowledge gaps.

**Conclusion:**

This review will provide an overview of existing evidence and identify gaps regarding the role of pharmacogenomics in psychotropic medication discontinuation. The findings will help to inform future research on the integration of pharmacogenomics into individualised tapering strategies.

## Introduction

Discontinuation of psychotropic medications presents a considerable and increasingly recognised clinical challenge ^1, 2^. Psychotropic medications typically include drugs that have effects on the central nervous system, with an influence on thinking, mood, consciousness, behaviour, or other mental processes. The term psychotropic medications generally encompasses a broad group of drugs, most commonly antidepressants, antipsychotics, mood stabilisers, anxiolytics, hypnotics, stimulants, and opioids ^3^. For patients treated with these medications, the process of dose reduction, cessation, or replacement can trigger withdrawal symptoms and/or, relapse of the underlying condition ^1, 2, 4, 5^. Withdrawal symptoms can range from mild and short-lived to severe and persistent ^6^. Initial withdrawal symptoms may include dizziness, headache, nausea, vomiting, irritability, anxiety, insomnia, vivid dreams, sensory disturbances, tremors, and in some cases, seizures. Protracted withdrawal symptoms may manifest as memory loss and concentration difficulties, hyperalgesia, and others ^6, 7^. However, considerable difficulties still exist in distinguishing symptoms of the original condition for which the medication was first prescribed and those induced by the discontinuation of the medication ^7, 8^. Furthermore, several factors influence the onset and course of withdrawal symptoms, including patient related factors (e.g. age and gender ^9, 10^, psychological factors ^11, 12^, and genetic predisposition ^13, 14^), and medication-related factors (e.g. type of medication ^7, 15– 17^, dosage and duration of medication use ^9, 11, 17^, and drug half-life ^7, 18^). Recognising these factors is crucial for prevention, early detection, and management of withdrawal symptoms.

A recent comprehensive systematic review on antidepressant treatment found that approximately one in three patients discontinuing these medications experience withdrawal symptoms, however, the frequency and intensity of these symptoms differ among antidepressant classes ^19^. Symptoms commonly emerge within a few days of dose reduction or cessation and are often transient, though in some cases they persist for several weeks or months ^18^. The onset and severity of discontinuation symptoms are strongly influenced by pharmacokinetic factors such as elimination half-life and metabolic rate. Antidepressants with short half-lives, such as paroxetine and venlafaxine, are associated with more frequent and severe withdrawal reactions compared with longer-acting drugs like fluoxetine ^18^. Differences in metabolism further contribute to variability, as several drugs depend on cytochrome P450 (CYP450) enzymes for elimination. Consequently, patients who are rapid metabolisers may experience faster drug clearance and more abrupt plasma concentration changes, leading to higher risk of withdrawal symptoms. For example, paroxetine, which is a substrate and strong inhibitor of CYP2D6 with a short half-life, may produce more severe discontinuation effects in rapid metabolisers than in normal or poor metabolisers ^20^.

To minimise withdrawal symptoms in patients taking psychotropic medications, clinical practice guidelines emphasise gradual dose reduction and patient education on the risks of abrupt cessation. For example, most guidelines recommend reducing antidepressant doses progressively over a period of at least four weeks ^18, 21, 22^. Similar strategies apply to the discontinuation of other psychotropic agents including antipsychotics ^23^, benzodiazepine and benzodiazepine receptor agonists ^24^, mood stabilisers ^25^, and opioids ^26^. Overall, gradual tapering of psychotropic medications is consistently associated with a lower incidence and severity of withdrawal symptoms compared with abrupt or rapid discontinuation, yet standardised uniform tapering protocols across drug classes are still lacking ^27^.

Pharmacogenomics examines the influence of gene polymorphisms on individual responses to medications including the risk of adverse effects, toxicity, or therapeutic failure ^28^. Pharmacogenomic testing has already been implemented in clinical practice primarily using two types of approaches: pre-emptive and reactive ^29^. The pre-emptive approach seeks to optimise drug therapy by testing patients for multiple pharmacogenetic variants before any specific pharmacotherapy is initiated, leading to more personalised genotype-guided prescription ^30^. Reactive pharmacogenomic testing is performed in response to a prescribing decision and typically involves single-gene analysis to guide an immediate therapeutic choice ^28^. However, because results are obtained after the medication is prescribed, this approach can delay treatment adjustments, pose safety risks, and is less efficient than pre-emptive testing ^28, 31^. Although pre-emptive pharmacogenomic testing presents greater challenges for routine implementation, it is generally considered a more desirable strategy than reactive testing ^31, 32^.

While pharmacogenomics has been extensively explored in relation to treatment efficacy and adverse drug reactions, its role in medication discontinuation and withdrawal symptoms remains insufficiently studied. Preliminary studies suggest that genetic variability may modulate susceptibility to withdrawal symptoms, implicating pharmacogenomic differences in interindividual variability during discontinuations ^13, 33^. Integrating pharmacogenomic insights into deprescribing and tapering strategies could therefore enhance the precision, safety, and personalisation of withdrawal management.

Accordingly, this scoping review aims to synthesise the available evidence on the role of pharmacogenomics in the discontinuation of psychotropic medication.

## Methods

Given that existing evidence on gene polymorphisms, tapering, and withdrawal outcomes is dispersed across diverse study designs and therapeutic contexts, a scoping review represents the most appropriate approach to systematically map and synthesise current knowledge. This methodology will allow for the inclusion of studies with diverse designs and outcomes, assessment of the available research, identification of key concepts, as well as knowledge gaps, thereby providing a foundation for future systematic reviews and evidence-based clinical recommendations.

The scoping review will be conducted in accordance with the Joanna Briggs Institute (JBI) methodology for scoping reviews and reported in accordance with the Preferred Reporting Items for Systematic Reviews and Meta-Analyses extension for Scoping Reviews (PRISMA-ScR) methodology ^34^. The adapted items of the PRISMA-P checklist for scoping reviews have been used in reporting this protocol ^35^.

### Scoping review question

The research question was developed in accordance with the Population Concept Context (PCC) framework ^36^:

### What is the available evidence regarding the role of pharmacogenomics (concept) on patients treated with psychotropic medications (population) in the process of the medication discontinuation (context)?

Population

The eligible population for this review is patients treated with psychotropic medications (antidepressants, antipsychotics, mood stabilisers, anxiolytics, hypnotics, stimulants, and opioid medications). No age restrictions will apply.

Concept

This scoping review will consider any evidence examining the role of pharmacogenomics in drug metabolism, transport, and receptor response (e.g., CYP enzymes, GABA receptors, *ABCB1*, *COMT*, *OPRM1*, *SLC6A4*, *HTR2A*, *BDNF*, and UGT enzymes) in the course of medication discontinuation.

Context

The review will include literature focusing on the process of medication discontinuation, including factors such as the duration and rate of tapering, the occurrence of withdrawal symptoms, and adverse drug reactions (ADRs) during dose reduction. Studies reporting on the success rate of discontinuation, relapse rates, or the need for treatment re-initiation will also be considered within the scope of this review.

## Defining and aligning the objectives and research question

This scoping review aims to map available evidence on the role of pharmacogenomics in the process of psychotropic medication discontinuation.

The objectives of this scoping review are to:

1. Map the scope and characteristics of existing literature describing pharmacogenomics associated with the process of discontinuing psychotropic medications, with specific attention to withdrawal symptoms, duration and rate of tapering, relapse rates, and discontinuation outcomes.
2. Summarise specific gene–drug or gene–drug class associations that have been investigated in relation to medication discontinuation and withdrawal symptoms.
3. Identify existing knowledge gaps and propose directions for future research on the role of pharmacogenomics in the discontinuation of psychotropic medication.

### Eligibility criteria

The eligibility criteria were defined according to the PCC framework, which ensures that the inclusion of studies is comprehensive and aligned with the objectives of the review. All study designs and evidence types relevant to the research question will be considered to capture the full extent of available knowledge. The inclusion and exclusion criteria are summarised in Table 1.

**Table 1.** Eligibility criteria for literature inclusion in the scoping review.

*Inclusion criteria*	*Exclusion criteria*
*Population* Patients treated with psychotropic medication (antidepressants, antipsychotics, anxiolytics, hypnotics, mood stabilisers, stimulants, opioids).	Patients not taking psychotropic medication.
*Concept* The role of pharmacogenomics in drug metabolism, transport, and receptor response (e.g., CYP enzymes, GABA receptors, *ABCB1*, *COMT*, *OPRM1*, *SLC6A4*, *HTR2A*, *BDNF*, and UGT enzymes) in the course of medication discontinuation.	Genetic variants not related to psychotropic medications.
*Context* The process of medication discontinuation including the duration and rate of tapering, withdrawal symptoms, relapse rates, etc.	Literature focused on initiation or response to psychotropic medications (as opposed to discontinuation).
*Type of studies* Systematic reviews RCTs, non-RCT, and pilot RCTs Observational studies Case-control studies Cohort studies (prospective and retrospective) Cross-sectional studies Qualitative studies Case studies	Opinion pieces Conference abstracts Study protocols Grey literature (e.g., theses, dissertations)

RCT – Randomised Controlled Trial

### Information sources

To carry out the search, a three-step process recommended by the JBI Manual for Evidence Synthesis ^34^ will be followed. An initial limited search of Web of Science was conducted to identify relevant articles, using keywords and MeSH terms derived from the PCC framework ( Table 2). The preliminary search strategy, developed in collaboration with a specialist librarian, has been completed for Web of Science. The search strategy will be further adapted for the following databases:

**Table 2.** Relevant search terms following the Population Concept Context (PCC) framework with keywords for Web of Science.

	POPULATION	CONCEPT	CONTEXT
Web of Science	Psychotropic drugs Psychiatric medications Antidepressants Antipsychotics Anxiolytics Hypnotics Mood stabilisers Neuroleptics Benzodiazepines Z-drugs Stimulants Opioids	Pharmacogenomics Pharmacogenetics Pharmacogenetic testing Genetic testing Genotyping Gene polymorphism Gene variant Single nucleotide polymorphism SNP CYP enzymes GABA receptor *ABCB1* *COMT* *OPRM1* *SLC6A4* *HTR2A* *BDNF* UGT enzymes Biomarker Metaboliser	Deprescribing Tapering Dose reduction Treatment cessation Drug discontinuation Medication discontinuation Drug withdrawal Withdrawal symptoms Rebound symptoms Abstinence syndrome

- MEDLINE
- CINAHL
- EMBASE
- PSYCINFO

The reference list of identified studies will be further reviewed for additional sources.

### Search strategy

All studies obtained from the literature searches will be imported into Covidence® ^37^, an online review management tool, to support systematic organisation, and screening. All duplicates will be removed. Each identified title will first be screened against the predefined inclusion and exclusion criteria ( Table 1), followed by assessment of abstracts and subsequently full-text articles. Two reviewers will perform the screening independently, and any discrepancies will be resolved through discussion with a third reviewer. Reasons for exclusion at the full-text stage will be documented. The search strategy for Web of Science is documented in Table 3.

**Table 3.** Search strategy for Web of Science.

Search terms relating to population (separated by Boolean operator ’OR’)	(agomelatine OR alfentanil OR alprazolam OR amisulpride OR amitriptyline OR antipsychotic* OR antidepressant* OR anxiolytic* OR aripiprazole OR atomoxetine OR benzodiazepine* OR brexpiprazole OR bromazepam OR buprenorphine OR bupropion OR buspirone OR carbamazepine OR cariprazine OR chlorpromazine OR citalopram OR clomipramine OR clonazepam OR clorazepate OR clozapine OR codeine OR desipramine OR desvenlafaxine OR diazepam OR doxepin OR duloxetine OR escitalopram OR fentanyl OR fluoxetine OR fluvoxamine OR flurazepam OR gabapentin OR haloperidol OR hypnotic* OR hydrocodone OR hydromorphone OR iloperidone OR imipramine OR lamotrigine OR levomethadone OR levomilnacipran OR lithium OR lorazepam OR lumateperone OR lurasidone OR methadone OR methylphenidate OR milnacipran OR midazolam OR mirtazapine OR "mood stabiliser*" OR "mood stabilizer*" OR morphine OR naltrexone OR neuroleptic* OR nitrazepam OR nortriptyline OR opioid* OR opiate* OR oxazepam OR oxcarbazepine OR oxycodone OR paliperidone OR paroxetine OR pimavanserin OR pregabalin OR quetiapine OR remifentanil OR risperidone OR "selective serotonin reuptake inhibitor*" OR "serotonin norepinephrine reuptake inhibitor*" OR SNRI* OR SSRI* OR sertraline OR stimulant* OR sufentanil OR sulpiride OR tapentadol OR temazepam OR "TCA*" OR topiramate OR tramadol OR triazolam OR trifluoperazine OR trimipramine OR valproate OR venlafaxine OR vilazodone OR vortioxetine OR "z drug*" OR ziprasidone OR zolpidem OR zopiclone OR ((psychiatric OR psychotropic) NEAR/2 (drug* OR medication* OR medicine* OR pharmaceutical* OR substance* OR therap*)))
Boolean operator	AND
Search terms relating to concept (separated by Boolean operator ’OR’)	("ABCB*" OR allele* OR "BDNF*" OR biomarker* OR "COMT*" OR "CYP*" OR "GABA receptor*" OR genotyp* OR "HTR2A*" OR metaboliser* OR metabolizer* OR "OPRM1*" OR "PGx*" OR pharmacogen* OR phenotype* OR "serotonin transporter*" OR "single nucleotide polymorphism*" OR "SLC6A4*" OR "SNP*" OR "UGT*" OR (gene* NEAR/2 (polymorphism* OR test* OR variant*)))
Boolean operator	AND
Search terms relating to context (separated by Boolean operator ’OR’)	(deprescrib* OR taper* OR ((abstinence OR discontinuation OR rebound OR withdrawal) NEAR/2 (symptom* OR syndrome*)) OR ((dose* OR drug* OR medication* OR medicine* OR pharmaceutical* OR substance* OR therap* OR treatment*) NEAR/2 (cessation OR discontinu* OR reduc* OR wean* OR withdraw*)))

The process of identification of studies will be illustrated using a PRISMA flow diagram adapted for scoping reviews – PRISMA-ScR flow diagram ^38^.

### Data charting

Data extraction from the included studies will be conducted by two reviewers using a predefined data extraction form. To ensure consistency and accuracy, a random 20% subset of the studies will be independently extracted by a third reviewer, and the results will be compared. The preliminary types of information that will be extracted are:

- Author(s)/Study citation
- Year of publication
- Origin/country of origin
- Aims/purpose
- Type of study
- Research design and methodology (e.g. type of genotyping, tapering)
- Population characteristics (type of pharmacotherapy)
- Sample size
- Investigated gene polymorphisms
- Description of medication discontinuation process (including withdrawal symptoms, duration and rate of tapering, ADE, success rate, etc.)
- Key findings related to scoping review questions

Any revisions of types of information during the extraction process will be documented and reported in the final scoping review.

Data relevant to the review objectives and research question will be extracted, including study characteristics (author, year, country, design), population details, type of pharmacotherapy, investigated gene polymorphisms, discontinuation process, and key findings.

Any discrepancies between reviewers during data extraction will be discussed and resolved through consensus, with input from additional reviewer from the research team if required.

### Critical appraisal

Given that this scoping review is intended to provide a broad overview of the available literature, critical appraisal of the included studies is not recommended by the current JBI guidance ^34^.

### Data analysis and synthesis

Key study characteristics and findings will be summarised in tabular form, with the table structure refined during the data extraction phase. A narrative synthesis will accompany the tables to provide an overview of the evidence concerning gene polymorphisms associated with the discontinuation of psychotropic medications, including the genes studied, medication classes, outcomes, and study designs. The analysis will focus on mapping and characterising the existing evidence rather than synthesising study results. Existing gaps in the literature and areas requiring further research will also be identified and highlighted.

## Discussion

Pharmacogenomic testing is increasingly recognised as a tool to optimise pharmacotherapy by reducing adverse effects, toxicity, improving treatment efficacy ^28^, and supporting the personalisation of therapy. However, the potential role of gene polymorphisms in influencing withdrawal symptoms and the tapering process remains poorly understood and underexplored. This represents a significant gap, particularly for psychotropic medication, where discontinuation is frequently complicated by variable patient responses.

This scoping review will address this gap by systematically mapping existing evidence on the role of pharmacogenomic factors in the discontinuation of psychotropic medication. The review will chart the characteristics of included studies, study designs, the investigated genes, medication classes, and reported outcomes. The findings will provide an overview of the current state of evidence, identify areas where research is lacking, and potentially establish a foundation for understanding how pharmacogenomic variation may contribute to interindividual differences in discontinuation outcomes. Ultimately, this review may inform future research aimed at integrating pharmacogenomic insights into individualised medication discontinuation strategies.

### Dissemination of findings

The completed review will be submitted for publication in a peer-reviewed journal.

## Data Availability

No underlying data are associated with this article. Open Science Framework: The role of pharmacogenomics in the discontinuation of psychotropic medication: a scoping review protocol. https://doi.org/10.17605/OSF.IO/EY7HM ^39^ The project contains the following extended data: PRISMA-ScR-Checklist_OSF_NS (PRISMA-P checklist) Search Strategy (Web of Science Search Strategy) PRISMA-P checklist for ‘The role of pharmacogenomics in the discontinuation of psychotropic medication: a scoping review protocol‘. https://doi.org/10.17605/OSF.IO/EY7HM ^39^ Data are available under the terms of the Creative Commons Zero "No rights reserved" data waiver (CC0 1.0 Public domain dedication).
