# Supplementary material for: The role of pharmacogenomics in the discontinuation of psychotropic medication: a scoping review protocol
Source: HRB Open Res. 2026 Feb 10;9:4. Originally published 2026 Jan 5. [Version 2] doi: 10.12688/hrbopenres.14314.2 (PMC12917353; doi:10.12688/hrbopenres.14314.2)
Supplement: Peer review history

# Reviewer report: Reviewer response for version 2

**DOI:** 10.21956/hrbopenres.15803.r53418

**Published:** 2026-02-18

**Authors:** Ramdas Bhat

**Affiliations:** 1 Pharmaceutical Sciences, Father Muller Charitable Institutions (Ringgold ID: 79611), Mangaluru, Karnataka, India

The authors have satisfactorily addressed the points raised during peer review. The rationale for maintaining a broad scope across psychotropic medication classes is now clearly justified, and the methodological clarifications strengthen the transparency and reproducibility of the protocol. Overall, the revisions improve clarity without altering the core objectives or design of the study. I have no further comments and support the finalisation of this protocol for indexing.

Is the study design appropriate for the research question?

Yes

Is the rationale for, and objectives of, the study clearly described?

Yes

Are sufficient details of the methods provided to allow replication by others?

Partly

Are the datasets clearly presented in a useable and accessible format?

Not applicable

Reviewer Expertise:

Neuropharmacology, psychopharmacology, pharmacogenomics, personalised medicine, variability in drug response, and psychotropic medication use and discontinuation.

I confirm that I have read this submission and believe that I have an appropriate level of expertise to confirm that it is of an acceptable scientific standard.

# Reviewer report: Reviewer response for version 1

**DOI:** 10.21956/hrbopenres.15756.r52689

**Published:** 2026-01-22

**Authors:** Malcolm Hopwood

**Affiliations:** 1 University of Melbourne, Melbourne, Australia

Thank you for the opportunity to review this interesting protocol paper and it is my opinion it is suitable for indexing. The authors primarily bring attention to the potential role  of pharmacogenomic factors in individual variation in psychotropic drug withdrawal. This an area of increasing clinical and research focus, all be it with considerable debate which the authors attest to. This issue had been most recently focused on the area of antidepressant withdrawal and its relative frequency and severity. The focus of the proposed review is thus a good one. 

 This does creates an interesting question as to why the authors have chosen to focus the scoping review on a quite broad range of psychotropic medications rather than just say antidepressants. It is certainly less common to overlap primarily analgesic psychotropic agents such as the opiates with antidepressants and anti-psychotics. In the end, the extent of current well developed study looking at the role of pharmacogenomics in withdrawal from any of these agents is likely relatively limited so a broad approach may be fine. 

 The objectives of the coping review are clearly stated and the method is well outlined and appropriate. The search strategy is clear as is the range of search terms including the likely psychotropic agents and various terms that might be used to describe withdrawal related processes. Importantly, I strongly support the decision not to undertake critical review at this point.

Is the study design appropriate for the research question?

Yes

Is the rationale for, and objectives of, the study clearly described?

Yes

Are sufficient details of the methods provided to allow replication by others?

Yes

Are the datasets clearly presented in a useable and accessible format?

Not applicable

Reviewer Expertise:

Mood Disorders, Pharmacology, Clinical trials, Pharmacogenomics

I confirm that I have read this submission and believe that I have an appropriate level of expertise to confirm that it is of an acceptable scientific standard.

# Response

**Published:** 2026-01-30

**Authors:** Natalia Stollarova

**Affiliations:** Comenius University in Bratislava Faculty of Pharmacy, Bratislava, Bratislava Region, Slovakia

We are very grateful to the reviewer for reviewing our manuscript and for the encouraging comments. We are delighted by the recognition of the relevance and timeliness of the proposed scoping review.

 In terms of the reviewer’s query regarding the broad range of psychotropic medications, we were conscious that limiting the focus of the review to certain drug classes could result in very limited findings. Therefore, we have purposefully decided to keep the scope of this review broad.

# Reviewer report: Reviewer response for version 1

**DOI:** 10.21956/hrbopenres.15756.r52690

**Published:** 2026-01-19

**Authors:** Ramdas Bhat, Murari Upadhay

**Affiliations:** 1 Pharmaceutical Sciences, Father Muller Charitable Institutions (Ringgold ID: 79611), Mangaluru, Karnataka, India

The paper describes a protocol of a scoping review which is aimed at undertaking the literature review of the role of pharmacogenomics in psychotropic medication discontinuation. The subject is applicable and timely, particularly considering that there is growing awareness regarding withdrawal symptoms, relapse, and individual differences during the discontinuation of psychotropic drugs. Pharmacogenomics is already widely utilized in the field of drugs selection and dose adjustment, but the area of pharmacogenomics in deprescribing and drug discontinuation remains poorly studied, and the given protocol attempts to fill the gap in a significant manner.

 The justification of the study is well stated. The background section is sufficient to describe the clinical issues related to the discontinuation of psychotropic drugs and presents a sensible argument as to why genetic differences can affect the discontinuation process. The aims of the review are clearly designed and logically developed out of the background. The application of the Population-Concept-Context framework to formulate the review question is suitable and assists in keeping the focus.

 The research question is appropriate to the overall study design. The scoping review is a valid option, as the topic under discussion is exploratory in nature, and the range of possible study designs, drug classes, and genetic markers is likely to be diverse. The aim to adhere to Joanna Briggs Institute methodology and report in line with PRISMA-ScR is suitable and demonstrates the knowledge of the best practices related to conducting scoping reviews. The choice of not conducting formal quality appraisal is agreeable to this kind of review.

 The methods section is generally clear, although there are parts that are described not fully and could be clarified. The search databases that it uses are suitable, and it includes an example search strategy that enhances transparency. Nonetheless, the protocol fails to explicitly state the time range of the literature search, e.g., the studies will be included in the database since its inception to a certain end date. This is to be brought out clearly. There is also no mention of language restrictions and it is not specified whether non-English studies are going to be included or not. This is something that should be clarified to eliminate confusion. Besides that, it is also mentioned in the protocol that grey literature will not be used, but the reasons behind this choice are not brought forth. Since the research in pharmacogenomics is dynamic, a concise explanation of why grey literature was not included would be a reinforcement to the methods section. Additional information on how the changes in the form of data charting would be addressed and reported throughout the review process would enhance reproducibility as well.

 Since this is a protocol, no datasets are given and consequently data presentation assessment is not possible. The authors rightfully report that they do not have any underlying data that are related to the article and offer such supplementary materials as the search strategy and reporting checklist via an open repository, which is useful in terms of transparency.

 In order to provide scientific validity of the protocol, the following aspects would be considered: clear-specification of the search time frame, explicit clarification on the inclusion or exclusion criteria based on language and justification of why grey literature would be excluded. Considering these would enhance better methodology but it would not alter the overall purpose of the study.

Is the study design appropriate for the research question?

Yes

Is the rationale for, and objectives of, the study clearly described?

Yes

Are sufficient details of the methods provided to allow replication by others?

Partly

Are the datasets clearly presented in a useable and accessible format?

Not applicable

Reviewer Expertise:

Neuropharmacology, psychopharmacology, pharmacogenomics, personalised medicine, variability in drug response, and psychotropic medication use and discontinuation.

We confirm that we have read this submission and believe that we have an appropriate level of expertise to confirm that it is of an acceptable scientific standard, however we have significant reservations, as outlined above.

# Response

**Published:** 2026-01-30

**Authors:** Natalia Stollarova

**Affiliations:** Comenius University in Bratislava Faculty of Pharmacy, Bratislava, Bratislava Region, Slovakia

We are very grateful to the reviewers for their comprehensive evaluation of our scoping review protocol, and for the feedback they have provided. We have responded to the relevant comments below and revised the manuscript accordingly. All revisions are visible using tracked changes.

1. Clarification of the search time frame.

Response: In line with the objectives of a scoping review, no date restrictions were applied in order to capture the full scope of available published and peer-reviewed evidence. We have now clarified this in the Methods section, and the table outlining the eligibility criteria.

 “In order to capture the full scope of relevant published and peer-reviewed evidence, no date or language restrictions will be applied.”

  

1. Explicit clarification on the inclusion or exclusion criteria based on language.

Response: No language restrictions will be applied, and this has now been explicitly stated in the Methods section and in the table outlining the eligibility criteria.

 “Studies published in languages other than English will be translated using professional translation tools where necessary.”

  

1. Justification of why grey literature would be excluded.

Response: We have now elaborated on the reason for excluding grey literature in the Methods section: 

 “Grey literature will be excluded due to practical and resource constraints and to ensure that the review focuses on primary research studies that have undergone peer review.”
